# Bringing the Public Health Informatics and Technology Workforce Together: The PHIAT Conference

**DOI:** 10.2196/55377

**Published:** 2024-06-11

**Authors:** April Moreno Arellano, Huan-ju Shih, Karmen S Williams

**Affiliations:** 1 Public Health Media Network San Diego, CA United States; 2 Health Administration and Policy College of Public Health George Mason University Fairfax, VA United States; 3 Department of Health Policy and Management Graduate School of Public Health and Health Policy City University of New York New York, NY United States

**Keywords:** public health informatics, health informatics, technology, health technology, digital health, digital intervention, digital interventions, conference, health conference, health conferences, public health workforce, public health worker, public health workers, PHIAT Conference, PHIAT, public health, health surveillance

## Abstract

The field of public health informatics has undergone significant evolution in recent years, and advancements in technology and its applications are imperative to address emerging public health challenges. Interdisciplinary approaches and training can assist with these challenges. In 2023, the inaugural Public Health Informatics and Technology (PHIAT) Conference was established as a hybrid 3-day conference at the University of California, San Diego, and online. The conference’s goal was to establish a forum for academics and public health organizations to discuss and tackle new opportunities and challenges in public health informatics and technology. This paper provides an overview of the quest for interest, speakers and topics, evaluations from the attendees, and lessons learned to be implemented in future conferences.

## Introduction

In recent decades, the field of public health informatics has undergone significant evolution, propelled by advancements in technology and the imperative to address emerging public health challenges. From its early beginnings in manual data collection to the widespread adoption of electronic health records and sophisticated data analytics, public health informatics has played a pivotal role in revolutionizing health care data management and surveillance systems. This historical trajectory underscores the continuous efforts to leverage technology and data-driven approaches to enhance public health outcomes and address the complex needs of populations worldwide. As the field continues to grow and adapt, understanding its historical context provides valuable insights into its current state and future directions. Especially after the COVID-19 pandemic, public health informatics has continued to grow as a field, and as expected, workforce needs have and will continue to expand for health information fields [1].

According to the US Bureau of Labor Statistics, health information technologists and medical registrars have a job growth outlook of 16% over the next 10 years, with an average of 3100 position openings per year [2]. This includes specialized positions in public health informatics and technology. However, growing and expanding does not come without workforce issues, such as recruitment, diversity, retention, burnout, and posttraumatic stress disorder among public health, public health informatics, and technology workers, which was especially exacerbated by COVID-19 [3-5]. In addition to these challenges, although there is rising enrollment in public health programs, there are fewer graduates entering public health agencies [6,7].

There are US-based conferences, such as the American Public Health (APHA) Association Annual Meeting [8], AcademyHealth [9], and other public health conferences [10-13], as well as a range of technology conferences, such as the Associates of Computing Machinery (ACM) [14] and Institute of Electrical and Electronics Engineers (IEEE) [15] conferences; there is also the American Medical Informatics Association (AMIA) [16] conference for biomedical and clinical informatics. Nevertheless, few conferences are available to address the bridge between public health, informatics, and technology.

In 2023, the inaugural Public Health Informatics and Technology (PHIAT) Conference was held as a 3-day hybrid conference. The PHIAT Conference occurred with an in-person option on the third day at the University of California, San Diego. The aim of the conference was to create an environment for academia and public health organizations to discuss and address emerging public health challenges and opportunities specifically in and with informatics and technology [17].

## Preliminary Research: Gathering Interest

At the time of the first PHIAT Conference, we had not identified any public-facing events directly focused on public health informatics and technology. To gather interest and ideas on how and where to announce the conference, we designed a preliminary survey to understand the feasibility of a conference dedicated specifically to public health informatics and technology. The survey was shared with the AMIA Public Health Informatics Working Group [18], the APHA LinkedIn group [19], and the Chronic Disease Geographic Information Systems Basecamp group of public health agency practitioners [20].

The preliminary survey included both closed- and open-ended questions related to interest in the idea of a public health IT conference, likelihood of attending a conference in June of the same year, rating of specific public health topics, preferred conference format, likelihood of attending the conference in person, and preferred level of participation. The brief survey was fielded from February 2 to 6, 2023, with most responses in the first 2 days.

This initial survey resulted in 46 responses and indicated a strong interest in the conference. Most of the participants had general positive reactions and indicated a willingness to pay for and attend the hybrid conference. The response rates for each question varied because not all respondents answered every question, some of the questions allowed respondents to answer with more than 1 choice, and all questions were optional.

**Figure 1:**
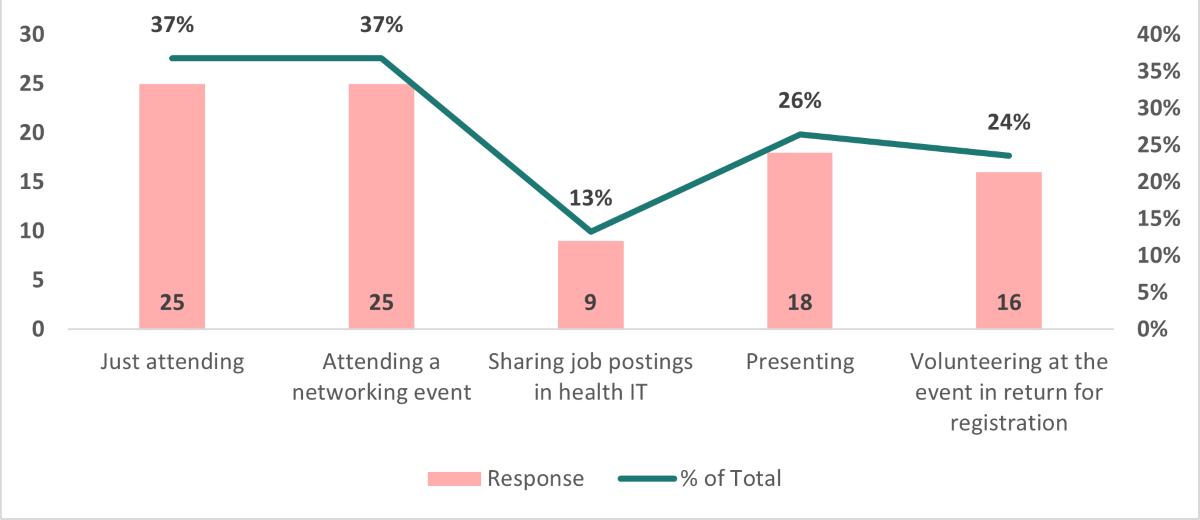
Interest in attending a public health IT conference.

The data presented in Figure 1 use a scale of 1 to 5 to denote interest level (where 5 signifies high interest and 1 signifies low interest) in a public health IT conference. Most of the respondents (23/42, 55%) noted a high level of interest. Additionally, 40% (17/42) of respondents were moderately interested. Importantly, none of the respondents expressed a lack of interest in the public health IT conference. These statistics underscore a substantial demand for hosting more public health IT conferences in the future.

**Figure 2:**
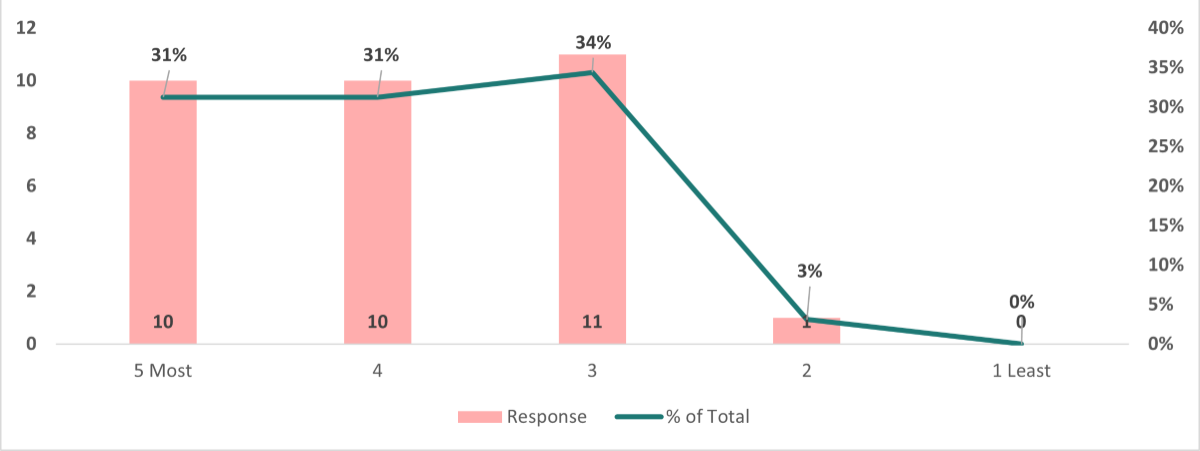
Likelihood of attending the conference in June 2023.

Conclusions regarding the likelihood of attending a conference months after this survey can be drawn from the data displayed in Figure 2. Using a scale of 1 to 5 to indicate interest levels (where 5 denotes strong interest and 1 denotes low interest), 34% of total responses showed a moderate degree of interest in attending the public health IT conference (11/32; n=14 missing values). In addition, 31% (10/32) gave the conference a rating of either 5 or 4 on the scale, demonstrating a strong level of interest. The significant relevance and interest respondents have expressed in attending the public health IT conference are illustrated in Figure 2, which supports the earlier findings.

**Figure 3:**
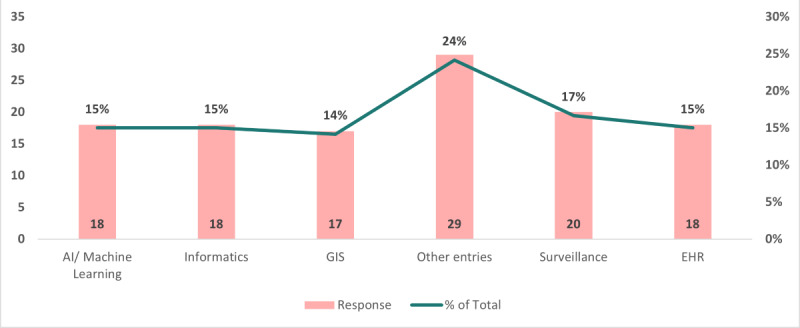
Preferred public health IT conference topics.

Figure 3 provides insights into the public health IT topics that piqued the interest of the respondents. These findings were valuable for this and future conference planning. The results indicated the following trends: the highest area of interest is categorized as “other entries,” such as public safety, alternatives to certain software, health information exchange and data sharing, and standards and guidance. Additionally, topics related to surveillance garnered significant attention, with 20 of 120 (17%) responses showing interest. The remaining topics garnered comparable levels of interest among respondents. Specifically, artificial intelligence (AI) and machine learning, electronic health records (EHRs), informatics, and geographic information systems (GISs) attracted similar levels of interest, each capturing 15% of the responses (18/120). Lastly, GISs received interest in 17 of 120 responses. These insights provide a comprehensive view of the preferences among the respondents for various public health IT topics.

**Figure 4:**
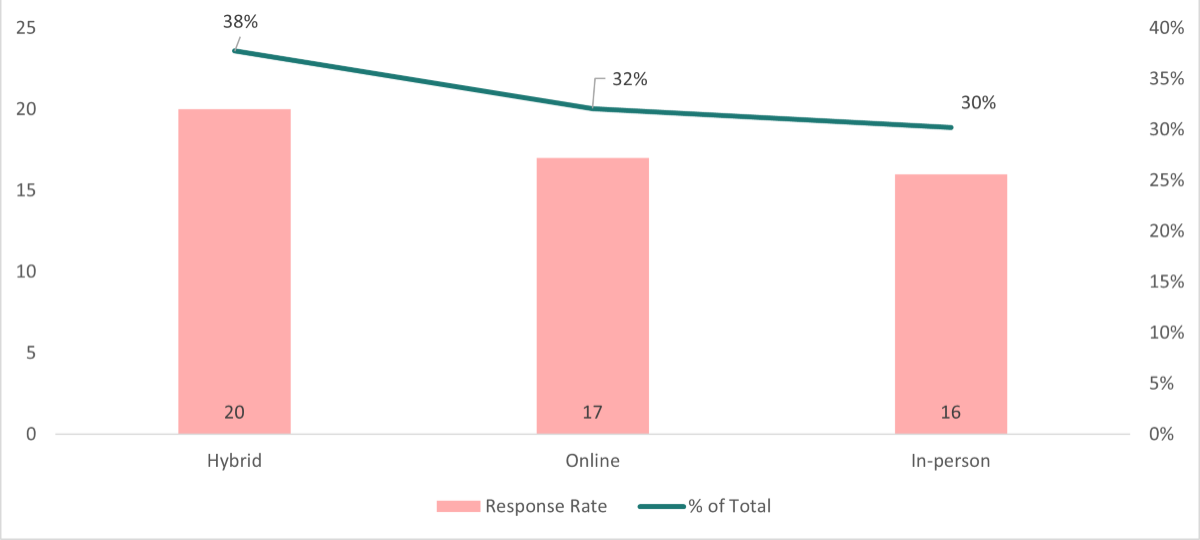
Preferred format for the conference.

Based on the data presented in Figure 4, valuable insights can be gleaned regarding the potential formats for future public health IT events after the COVID-19 pandemic. These insights could be instrumental in guiding decisions on how to attract a greater number of participants while maintaining optimal learning effectiveness and efficiently budgeting the conference costs. Among the 53 responses, the preferred formats for a public health IT conference were as follows: 20 responses (38%) indicated a preference for a hybrid format; 17 responses (32%) expressed a preference for a web-based format; and 16 responses (30%) favored an in-person format. These findings provided a clearer understanding of the preferences of respondents regarding the format of the public health IT conference, thereby informing future planning and decision-making processes.

**Figure 5:**
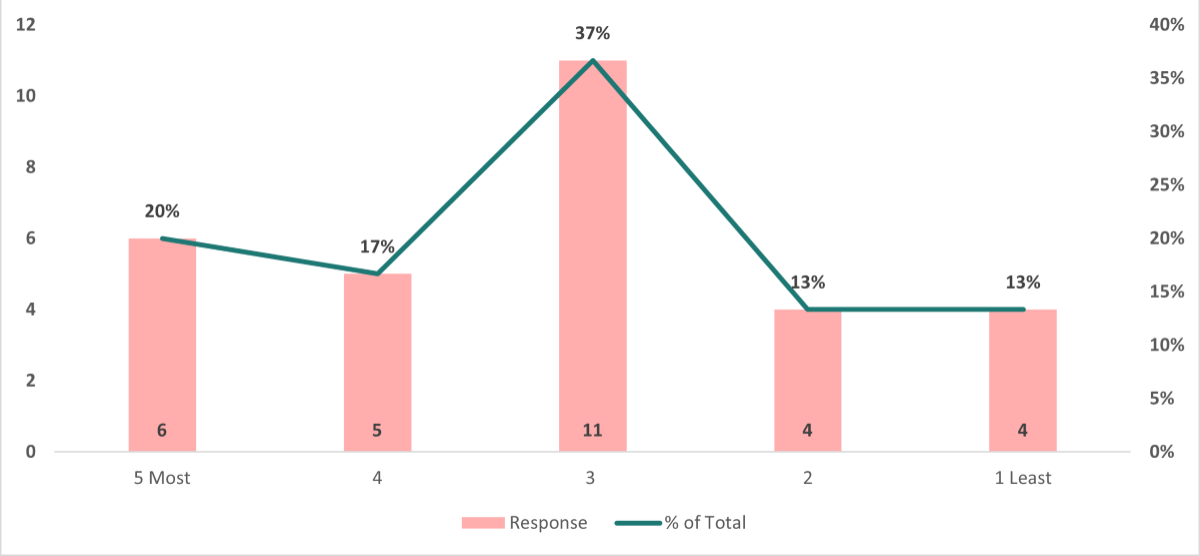
Likelihood of in-person attendance in San Diego, CA.

We extended our inquiry by posing a follow-up question based on the previous questions. Figure 5 offers valuable insights into the anticipated participation levels in public health IT events after COVID-19. Over time, this information will facilitate the observation of growth trends in the public health informatics IT conference landscape. Among the 30 total respondents, a majority, 11 individuals (37%), expressed their intention to participate in the conference. Notably, 6 respondents (20%) displayed a high likelihood of joining the conference in person in San Diego, California. Based on the groups that were selected to have access to the survey, these results provide a view of respondents’ intentions to participate, aiding in gauging interest and contributing to future conference planning endeavors.

**Figure 6:**
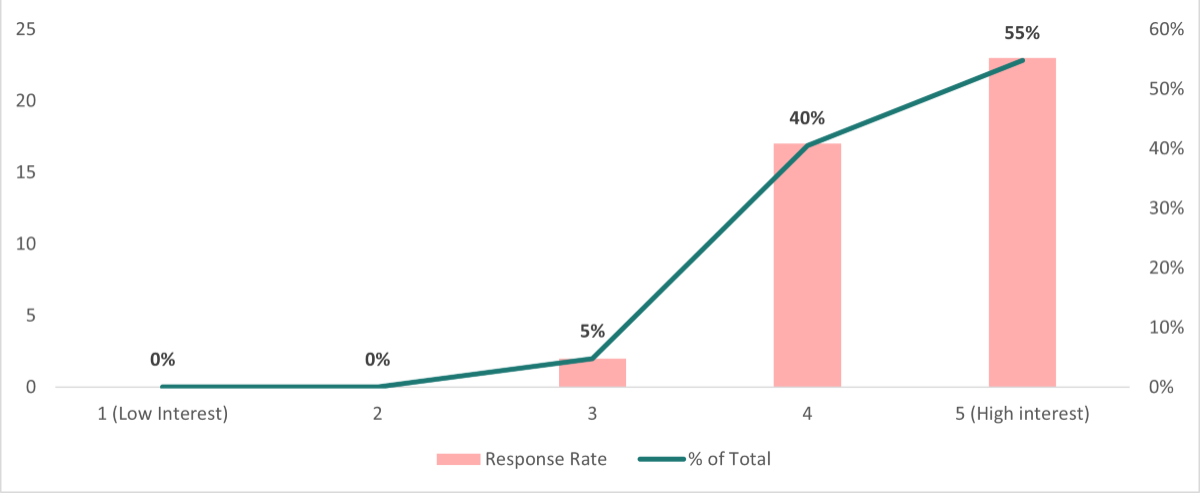
Preferred levels of participation.

We polled various public health and technology interest groups to learn about their preferences and interests to further increase the value of a public health IT conference. Five different categories were clarified by the insights (Figure 6). These insights are crucial for tailoring the conference to meet the preferences of the participants. The survey outcomes highlighted the following preferences among the 68 responses: the most popular choice, preferred by 20 individuals (29%), was attending a networking event. Attending the conference exclusively was favored by 16 respondents (24%); 13 respondents (19%) expressed their interest in volunteering at the event in exchange for registration. Additionally, 12 individuals (18%) indicated their desire to present at the conference. Lastly, 7 respondents (10%) expressed interest in sharing job postings during the conference. These findings offer a comprehensive understanding of the attendees’ preferences and aspirations, which will contribute to creating a well-rounded and engaging public health IT conference.

## Description of the Conference

### Cross-Sector Collaborations

The PHIAT Conference was designed to include the latest academic research on public health informatics and technology. The event featured public health department representatives, academic researchers, and businesses sharing their innovative approaches to improving public health. We enjoyed providing this space for interaction and communication across these various sectors and look forward to continuing to cultivate these discussions to share public health informatics and technology best practices, policy implementations, case studies, and the latest innovative developments in the field.

### Structure of the Conference

The hybrid nature of the conference allowed for academics, organization professionals, and students to attend either via video conferencing or in-person for the day 3 workshops during Pacific Standard Time. We found that this maximized the opportunity for individuals to be able to attend across demographic, socioeconomic, and geographic spaces where time, location, or cost were potentially constraining factors. The option of meeting either online or in person provided more opportunities for internationally based individuals to attend our conference. For example, speakers from other continents were able to present, sleep, and watch the rest of the event online without having to travel. Some were able to share prerecorded presentations and to participate online at a time-zone appropriate time, watch the recording, connect with other speakers, and submit their event feedback later. Additionally, students were able to register at a discounted rate and attend the event remotely if travel funding was a concern. Finally, attendees with logistical challenges were able to attend live for a short period of time, leave, and then return without the need to travel to the event.

Some presenters, such as the national coordinator for health IT and the presenters from Esri Health and Human Services and the Department of Biomedical Informatics of the University of California, San Diego, were personally invited to speak, due to their known expertise and our familiarity with their work. Additionally, many of the presenters were selected through their abstract submissions, quality of presentation, and suitability for the theme of the conference. A team of 5 individuals selected the abstracts, and talks were scheduled based on the theme of the day and time availability of speakers. In cases where the time zones were many hours apart, such as in Australia, presenters were given the opportunity to prerecord their sessions.

## Event Overview

The first day of the event included a range of discussions, such as community solutions, AI, machine learning, data sharing, and health equity. There were a variety of organizations and teams represented, such as universities, health centers, community-based agencies, and researchers.

We began the event (Multimedia Appendix 1 includes a link to the agenda) with a presentation led by KSW on the status of the public health informatics workforce and workforce needs now and in the near future. We continued the event with many of our featured invited presenters. The national coordinator for health IT, Micky Tripathi, PhD, MPP, discussed the US federal health IT strategy and goals for interoperable infrastructure under TEFCA (Trusted Exchange Framework and Common Agreement), emphasizing further collaborations with qualified health information networks (QHINs).

As Nanette Star, MPH, of Esri’s health and human services team mentioned on day 2 in her keynote speech, “pictures are worth a thousand words and maps are worth a thousand pictures.” Her keynote speech was focused on understanding health GISs to answer questions, in addition to sharing some use cases for GISs in public health. Additionally, on day 2 AMA provided a talk on developing strategy for GIS leadership, including important points such as understanding why the map is made, who it is made for, and what is seen and remains unseen in the data provided.

The event provided space for discussion on emerging themes of blockchain technology for public health data security, presented by Tsung-ting Tim Kuo, PhD, of the University of California, San Diego, biomedical informatics department, who shared some of his National Institutes of Health–funded research on blockchain models for health. Findings from opioid-related disorder research through natural language processing for social media and GISs for health were presented by Anthony Corso, PhD, of California Baptist University.

A broader discussion on technology and innovation for public health was provided by Azizi Seixas, PhD, of the University of Miami Media Innovation Lab. The organizers also led a discussion on public health big data, AI, and ethical challenges, with great insight from attendees. We were very aware that many of our speakers did not have specific backgrounds in public health. Their topics were intentionally welcomed due to their importance and relevance for the possibilities of public health innovation, collaboration, adaptation, and framework development. They were also invited because public health professionals often have not yet been working with these topics.

On the final day of the event, we met in person at the campus of the University of California, San Diego, where we provided 2 workshops that were also available via video conferencing. One workshop was hosted by Ming Hsiang Tsou, PhD, of San Diego State University on big data and GISs for precision public health. This was followed by a workshop on artifact evaluation by Gondy Leroy, PhD, of the University of Arizona, followed by health informatics presentations from researchers at the University of California, San Diego, and a networking reception where participants had the opportunity to discuss their conference reflections, build new partnerships, and share their interest in the future conference next year.

## Postconference Feedback

We conducted a postconference survey to gather insights from attendees’ experience and feedback, aiming to better understand their preferences and optimize our marketing strategies for the PHIAT Conference in the future. Most of the respondents learned about this conference via email and word of mouth and from the Public Health Podcast Network [21] newsletters and monthly events. Attendees of this inaugural conference were drawn to it for the opportunity to advance their professional development.

The postconference survey asked why participants were interested in attending; their comments, with our responses, are shown in Table 1.

**Table 1:** Postconference survey comments from participants and our responses.

Comment	Response
“I’m a public health informatics epidemiologist and I am really interested to learn more about how we can make our informatics program more advanced and efficient.”	PHIAT was designed with the public health workforce and academia in mind. With innovation and corporate development and academic partnerships, the goal was to improve the quality of public health data processes while fueling innovative ideas for research and development for positive public health implications.
“My position is focused on chronic disease informatics, and I was hoping to see examples of informatics methods and case studies using electronic health records or clinical data.”	Developing workflows for electronic health data and building health information exchange infrastructure are continuing goals for public health, and another goal with the PHIAT Conference is to cultivate better processes for building public health informatics infrastructures. The use of informatics is still relatively new for public health departments, and this is often beyond the scope of departmental epidemiological methods. We hope to include more public health informatics professionals to speak at our conferences as the event becomes more established.
“I was very interested in hearing from guest speakers experienced in the IT side of public health and knew there would be a great variety in professionals sharing their knowledge.”	There is a small percentage of public health professionals who have been trained in foundational IT skills, such as database development, the software development life cycle, and informatics frameworks, and we were excited to invite and bring these individuals together through the conference. We also intended to welcome new professionals and researchers into this field, either for their own research or professional development interests or to increase institutional knowledge at their public health departments.
“The main reasons for attending this conference were to foster and enhance professional growth and development. This public health informatics conference provides educational sessions (what tools can be used by combining machine learning and GIS), training workshops (how to develop a research question), and tutorials that focus on enhancing technical skills, understanding policy frameworks, and mastering health information technologies. Attending these meetings has allowed me to extend my horizons and keep ahead of the curve in my career.”	The decision not to focus on one specific public health technological topic, such as epidemiological surveillance, and to focus on a broad range of topics in informatics and technology for public health professionals provided the opportunity for professionals to learn from a breadth and depth of knowledge. We hope that professionals had the opportunity to learn about ideas, topics, and developments that they had not heard of before. We also hope that attendees left the event with new ideas, approaches, and concepts to advance the quality of their work and their career development.

Respondents indicated an interest in a variety of educational seminars, training workshops, and tutorials at the conference with the goal of improving their personal technical proficiency, comprehending policy frameworks, and mastering health information technologies. They expressed a desire to learn more about developing research questions, using electronic health records and clinical data in informatics approaches and case studies, and merging machine learning and GIS technologies, which were topics covered in our conference sessions. One of the respondents said, “Attending these meetings helped me stay at the forefront of the field as a public health informatics epidemiologist with a focus on chronic illness informatics.” In order to progress and refine their organization’s informatics program for increased efficiency and effectiveness, attending meaningful conferences has been crucial. One participant indicated that they learned about the conference from other guest speakers well-versed in the IT side of public health, which is driven by an insatiable need for information. They excitedly embraced the chance to receive insightful knowledge and immerse themselves in a wide range of skills.

Attendee feedback highlighted several key aspects of their experience at the public health informatics conference. They commended Nanette Star’s presentation and the use of QR codes in the slides to enhance interactivity. The suggestion to include more presentations of this nature for future events was well received. Additionally, attendees expressed interest in hearing from major health care organizations regarding their use of community health care data and the impact of IT developments on care improvements. In relation to the timing of the conference, we received suggestions that the length of the conference remain 1-3 days with 4-5 hours a day of presentations and 3 hours for practice, questions, and discussions.

In terms of cost, based on the 3 day–long conference in San Diego, which included a valuable networking reception, participants suggested an average registration fee of US $400 per person. In addition, based on the financial considerations that some attendees might have, participants also sought opportunities for a discounted student rate and a reduced rate for professionals currently experiencing unemployment. Further suggestions included hiring professional teams to run the event, which would help us focus on moderating the panels. In order to enhance the conference experience, attendees also suggested adding interactive sessions with explicit discussion questions or expert panels. They observed that several themes appeared to be more concerned with public health than public health informatics, which suggests that the conference’s thematic substance can move toward becoming more focused on public health informatics technologies. Partnering with recognized public health informatics leaders or organizations, like the US Centers for Disease Control and Prevention’s Office of Informatics and Information Resources Management, was also advised.

Overall, there was general interest in the various topics of public health technology and precision public health and enthusiasm in the discussions.

## Conclusion

The inaugural PHIAT Conference was an engaging, informative, and much-needed event providing public health professionals with information that many in the field were not previously familiar with. The technological side of public health is a crucial part of the future of health, yet many professionals are still new to the concepts of how AI, machine learning, and other technologies of precision public health can contribute to their organizations’ data and infrastructure capabilities.

## Lessons Learned

We found a high level of interest in the topics presented and are looking forward to continuing these discussions to further build, support, and develop public health technological infrastructure. In addition, most attendees appreciated the interactive sessions with discussion questions and expert panels, suggesting that more such sessions be included in future events.

When asked in the postevent survey about their feedback for improvement, attendees provided their feedback, including the statements in Table 1.

We plan to continue the PHIAT Conference yearly and collaborate with additional institutions who work in public health informatics. At this time, we will continue to host the event in the San Diego or southern California area.

## Future Plans

For the time being, the conference will be presented in 2 formats in alternate years. For 2024 and even-numbered years, the event will be a 1-day PHIAT summit event to continue with updates in the field by selected leaders in health IT, GISs, machine learning, modeling and simulation, and more to provide interim updates from policy and innovation perspectives. The event will return in odd-numbered years in the 3-day abstract submission format, highlighting new developments in various public health IT and informatics topics with discussion from academics, public health practitioners, and enterprises. To support event accessibility, we will likely continue to use an in-person, 3-day format for the next event, with the option to participate online as well for those who are unable to travel due to logistical or financial concerns. With the expansion of fellowships and programs in public health informatics and technology, we would like to collaborate and partner for a larger discussion on workforce preparedness. Additionally, we are seeking sponsorships to create a space for greater impact and reach in public health informatics and technology.

## Appendix

Multimedia Appendix 1 The 2023 Public Health Informatics and Technology Conference Agenda Booklet.

## Abbreviations

AI: artificial intelligence
EHR: electronic health record
GIS: geographic information system
ML: machine learning
PHIAT: Public Health Informatics and Technology
QHIN: qualified health information network
TEFCA: Trusted Exchange Framework and Common Agreement
