# Supplementary material for: Bringing the Public Health Informatics and Technology Workforce Together: The PHIAT Conference
Source: Online J Public Health Inform. 2024 Jun 11;16:e55377. doi: 10.2196/55377 (PMC11259087; doi:10.2196/55377)
Supplement: Multimedia Appendix 1 [file ojphi_v16i1e55377_app1.pdf]

2023 PUBLIC HEALTH INFORMATICS AND TECHNOLOGY CONFERENCE

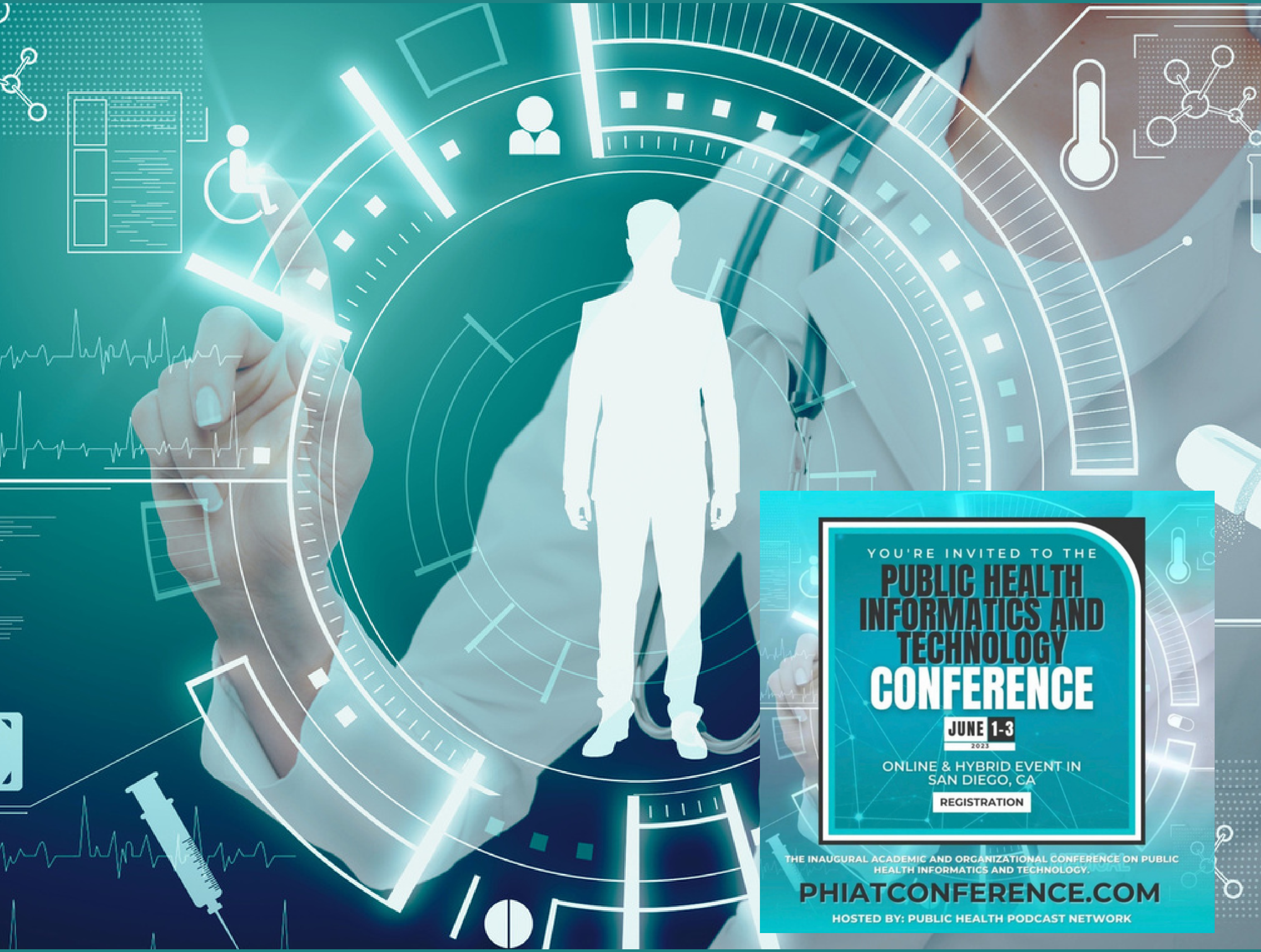

JUNE 1-3, 2023

Virtual and Hybrid at UC San Diego

PRESENTED BY PUBLIC HEALTH PODCAST AND MEDIA NETWORK

2023 PUBLIC HEALTH INFORMATICS AND TECHNOLOGY CONFERENCE

Welcome to the 2023 Public Health Informatics and Technology Conference. We are so excited that you chose to join us on this exploration of various topics related to public health, technology, and social determinants of health.

This conference was organized as an academic and public health organizational event addressing emerging public health challenges and opportunities in informatics and technology.

The conference organizers, April Moreno PhD and professor/collaborator Karmen Williams DrPH, stated that "As public health informatics doctoral researchers and professionals, we realized that public health needed its own informatics and Health IT conference. So, we decided to begin facilitating these discussions in the context of public health and the social determinants of health."

We are excited that you have joined us for this inaugural PHIAT Conference, June 1-3, 2023 both virtually and hybrid in San Diego, CA.

Thank you for attending.

Sincerely,

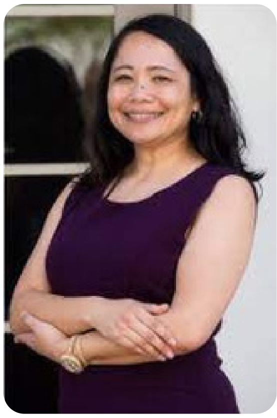

April Moreno, PhD
Public Health Podcast and Media Network

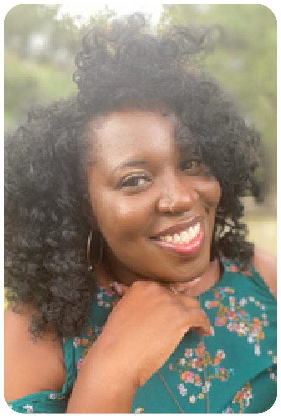

Karmen S. Williams, DrPH
City University of New York

PUBLIC HEALTH INFORMATICS AND TECHNOLOGY CONFERENCE 2023

PHIATCONFERENCE.COM

CONFERENCE SCHEDULE

DAY 1 - THURSDAY, JUNE 1, 2023

PACIFIC TIME (PT)	
9:00 - 9:30 AM	*Opening Session* - Karmen S. Williams, DrPH<br>**Welcome and Status of PHIAT**
9:30 - 10:30 AM	*Micky Tripathi*, PhD, MPP, ONC HIT **(KEYNOTE SPEAKER)**<br>**Updates from the National Coordinator for Health IT**
10:30 - 10:55 AM	*Angele Russell*, Colorectal Cancer Alliance (CCAlliance)<br>**Community-Driven Support Platform, BlueHQ: An Innovative Solution to Address CRC Patients' Needs**
10:55 - 11:05 AM	BREAK
11:05 - 11:30 AM	*Mohammed Bin Ahmed and Team*, Versante Health<br>**Reducing Stigma Across the HIV/STD-infected Communities and Reassuring Continuity of Care with AI-driven Solutions**
11:30 - 12:00 PM	*Ronald Lorenzo*, PhD, Prairie View A&M University<br>**The Convergence of Social Movements: Social Justice, Open Science, and Community Science: the Challenges, Opportunities of Entering the Public Conversation**
12:00 - 12:30 PM	*Shamsa Majid Lootah and Team*, Emirates Health Services<br>**AI-Driven Communicable Disease Management**
12:30 - 1:00 PM	Lunch/Networking Break
1:00 - 1:30 PM	*Tsung-ting Kuo*, PhD, UC San Diego Biomedical Informatics<br>**Blockchain for COVID-19 Data Sharing**
1:30 - 2:00 PM	*Joshua Bakasa and Team*, Clinton Health Kenya<br>**Leveraging Big Data and Machine Learning Technology for Effective Program Management to Improve Immunization Outcomes in Kenya**
2:00 - 3:00 PM	*Anthony Corso*, PhD, California Baptist University<br>**Using Location Analytics and NLP to Address the Opioid Crisis**
3:00 - 4:00 PM	*Irene Dankwa Mullan*, MD, MPH, Marti Health<br>**AI, Big Data, and Health Equity for Public Health**

PUBLIC HEALTH INFORMATICS AND TECHNOLOGY CONFERENCE 2023

PHIATCONFERENCE.COM

CONFERENCE SCHEDULE

DAY 2 - FRIDAY, JUNE 2, 2023

PACIFIC TIME (PT)	
9:00 - 9:30 AM	*Opening Session* - *April Moreno, PhD*<br>**Public Health GIS Strategy for Effective Leadership**
9:30 - 10:30 AM	*Nanette Star*, ESRI **(KEYNOTE)**<br>**Advancing Health & Human Services with GIS**
10:30 - 10:55 AM	*Rebecca Hogbin*, New South Wales Health, Australia<br>**Creating Smart Public Health Systems with REDCap and R: Lessons Learned from an Australian Country Public Health Unit**
10:55 - 11:05 AM	BREAK
11:05 - 11:30 AM	*Katie Allen*, Regenstrief Institute, Inc.<br>**Enhancing the Nation's Public Health Information Infrastructure: Moving Beyond the Pandemic**
11:30 - 12:00 PM	Conference Organizers, Karmen Williams and April Moreno<br>**Discussion on Public Health Big Data, AI, and Ethical Challenges**
12:00 - 1:00 PM	**Lunch Workshop**: *Corina Chung*, County of San Mateo<br>**Creating Efficient ArcGIS Online Web Mapping Application Workflows for Public Health Surveillance**
1:00 - 1:30 PM	*Azizi Seixas*, PhD, University of Miami **(KEYNOTE)**<br>**Technology and Innovation for Public Health**
1:30 - 2:00 PM	*Nancy Kinyua and Team*, Clinton Health Kenya<br>**A Geospatial Approach to Delineating Population Catchments and Defaulter Tracking for Pentavalent Vaccines in Kitui County, Kenya**
2:00 - 3:00 PM	*Josh Morgan*, PsyD, SAS<br>**Leveraging Whole Person Data Integration Across Public Organizations**
3:00 - 3:30 PM	*Craig Newman*, Altarum<br>**The Application of FHIR Interoperability Standard to Address Public Health Data Sharing Pain**
3:30 - 4:30 PM	*Kayla Jones, Jeffrey Duncan, State of Michigan*<br>**Evaluating the Use of SMART-on-FHIR to Improve Birth Certificate Data Quality in Michigan**
4:00 - 5:00 PM	NETWORKING DISCUSSION SPACE

PUBLIC HEALTH INFORMATICS AND TECHNOLOGY CONFERENCE 2023

PHIATCONFERENCE.COM

CONFERENCE SCHEDULE

DAY 3 - SATURDAY, JUNE 3, 2023

PACIFIC TIME (PT) at UC San Diego *online and in-person*

10:00 - 11:00 AM	**Workshop 1 - Big Data and GIS for Public Health** (Virtual)<br>*Ming-Hsiang Tsou*, PhD, San Diego State University
11:00 - 12:00 PM	**Workshop 2 - Evaluating Your Public Health IT Artifact**<br>*Gondy Leroy*, PhD, Arizona State University
**12:00 - 3:00 PM**	**NETWORKING LUNCH**
12:10 - 12:35 PM	*Ivan Copado*, University of California San Diego<br>**EHR Analysis for Vision Health of Refugee Populations in San Diego County**
12:35-12:55 PM	*Manreet Brar*, University of California San Diego<br>**EHR and Demographic Data for Analyzing Barriers to Medication Adherence Among Glaucoma Patients**
1:00 - 3:00 PM	NETWORKING

PHIAT Conference Evaluation

We invite your feedback on the conference, topics, and improvements for future conferences. Thank you again for attending!

https://shorturl.at/giloR

INVITED GUEST SPEAKERS

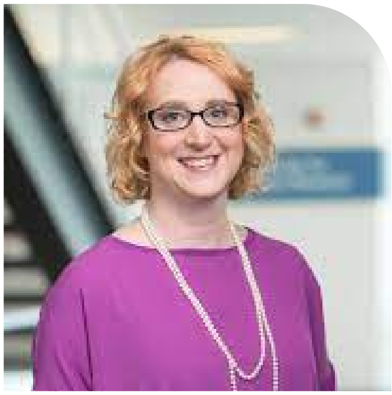

KATIE ALLEN, BS

Enhancing the Nation's Public Health Information Infrastructure: Moving Beyond the Pandemic

Katie Allen has been a part of Regenstrief Institute since 2008, when she joined the Clem McDonald Center for Biomedical Informatics. In 2021, she assumed the role of Data Scientist within the Public Health Informatics program. Katie is also a PhD candidate in Health Policy and Management at the Richard M. Fairbanks School of Public Health. Her dissertation focuses on barriers to trust in electronic health record derived social factors data. Katie's current role as a Data Scientist allows her to collaborate with investigators to advance their data-driven research agendas. Her current project-specific activities include incorporation of social determinants with clinical data, both area-level measurements and utilizing clinical text to identify person-level measurements, public health infrastructure and surveillance, and analyses examining the role of social factors in specific conditions.

ANTHONY CORSO, PHD

Using Location Analytics and NLP to Address the Opioid Crisis

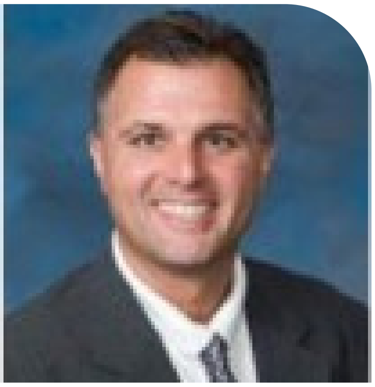

Dr. Corso joined California Baptist University in Fall 2006 as an adjunct faculty member in the discipline of Computer Information Systems. He held the position until August 2008 when hired as a full-time tenure-track faculty member. Dr. Corso is an AWS Certified Engineer as of February 2022.

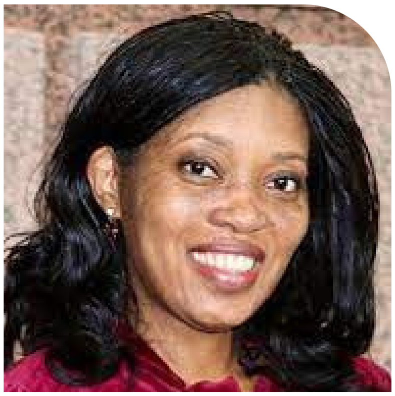

Irene Dankwa-Mullan, MD MPH

AI, Big Data, and Health Equity for Public Health

As the CMO and CHO of Marti Health, "Dr. Irene Dankwa-Mullan is a nationally recognized industry physician, thought leader, health equity scholar with over 20 years of diverse regional and national leadership experience in primary care, public health research, and industry. She is currently on the executive leadership team as a strategic advisor and Chief Health Officer at a health tech start-up, Marti Health. Marti Health is building a community-based management services organization, in collaboration with patient-centered medical homes and community organizations to address and improve healthcare inequities related to access to care, delivery of care, quality of care and cost of care. She was formerly Chief Health Equity Officer, IBM Watson Health (now Merative). She holds an adjunct professor position at the George Washington University Milken Institute School of Public Health. Dr. Dankwa-Mullan was formerly Director for extramural scientific programs at NIMHD, NIH. While at the NIH, she was active on several key strategic boards and committees, including many that were cross-sectoral and agency-wide, promoting health in all policies. She was awarded the NIH Director's Award for exceptional contribution to advancing the science of health disparities research. She is a steering committee member of the Coalition for Health AI that includes of subject matter experts from leading academic medical centers to ensure responsible use of AI in healthcare. She also serves on various advisory committees to promote public health, health equity, as well as on health tech start-ups working to address efforts impacting health disparities. Dr. Dankwa-Mullan has published widely on health disparities, including on the integration of health equity, artificial intelligence and machine-learning, ethical AI and social justice principles into data science methods and technology development lifecycle."

INVITED GUEST SPEAKERS

TSUNG-TING KUO, PHD

Blockchain for COVID 19 Data Sharing

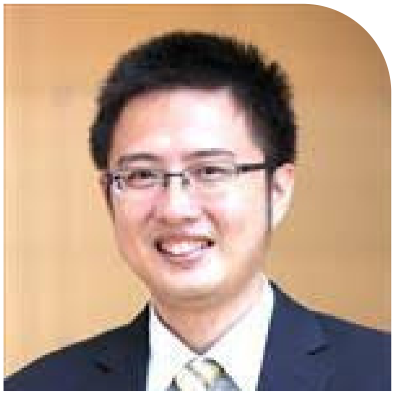

Dr. Tsung-Ting Kuo is an Assistant Professor of Medicine in University of California San Diego (UCSD) Health Department of Biomedical Informatics (DBMI). He earned his PhD from National Taiwan University (NTU) in the Institute of Networking and Multimedia. Prior to becoming a faculty member, he was a Postdoctoral Scholar at UCSD DBMI and received the UCSD Chancellor's Outstanding Postdoctoral Scholar Award. He was a major contributor towards the UCSD DBMI team winning the Office of the National Coordinator for Health Information Technology (ONC) healthcare blockchain challenge, and also the NTU team winning the Association for Computing Machinery (ACM) Knowledge Discovery and Data Mining (KDD) Cup competition four times. He was awarded a NIH R01 Research Project Grant, a K99/R00 Pathway to Independence Award with an Administrative Supplement, a R13 Support for Conferences and Scientific Meetings Grant, as well as UCSD Academic Senate Health Science Research Grants, for blockchain-based biomedical, healthcare and genomic studies. His research focuses on blockchain technologies, machine learning, and natural language processing.

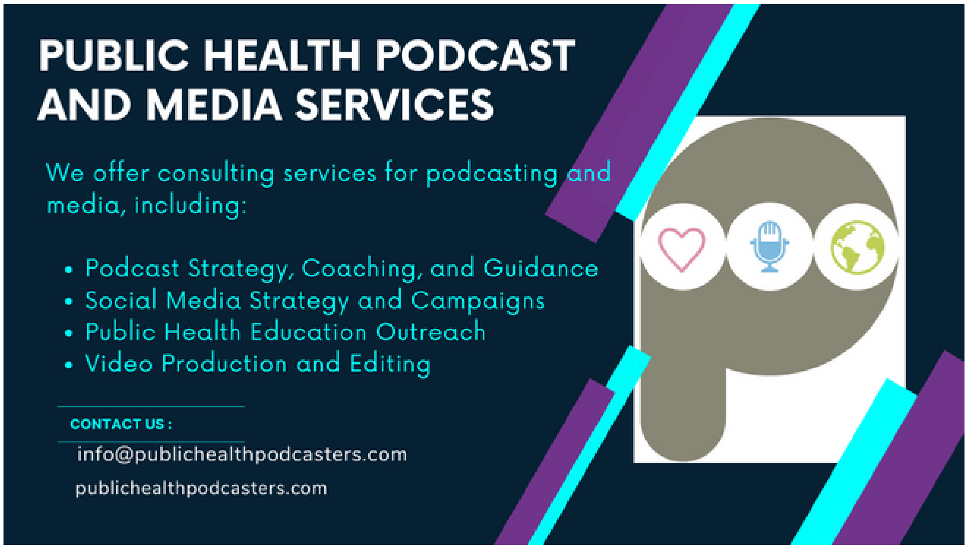

INVITED GUEST SPEAKERS

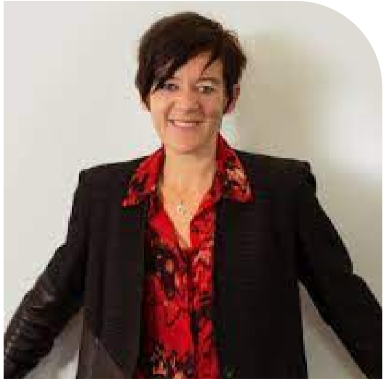

GONDY LEROY, PHD

Evaluating Your Health IT Artifact

Gondy Leroy, Ph.D., is Professor in MIS and Associate Dean for Research at the University of Arizona. Her research focuses on the design, development, and evaluation of information systems. Since graduating in 2003, she has worked on apps to facilitate communication with children with autism, search engines for biomedical information, and interview systems for crime witnesses. Her research focuses on natural language processing (NLP) and machine learning (ML) applied to projects with a practical and positive impact. She has won grants from NIH (NLM and NIMH), AHRQ, NSF, Microsoft Research, and several foundations, totaling more than $5.7M as principal investigator and another $2M on which she was a co-investigator. She earned a combined BS and MS (1996) in cognitive, experimental psychology from the Catholic University of Leuven (1996) in Belgium and a MS (1999) and Ph.D. (2003) in management information systems from the University of Arizona. She serves on the editorial board of the Journal of Database Management, International Journal of Social and Organizational Dynamics in IT, Health Systems, Journal of Business Analytics, and co-chairs several sessions, tracks, workshops, and conferences focusing on design science and healthcare IT. She is the author of the book “Designing User Studies in Informatics (Springer, 2011). Finally, she is an active contributor to increasing diversity and inclusion in computing and founded and leads the “Tomorrow’s Leaders Equipped for Diversity” program at the University of Arizona’s Eller School of Management.

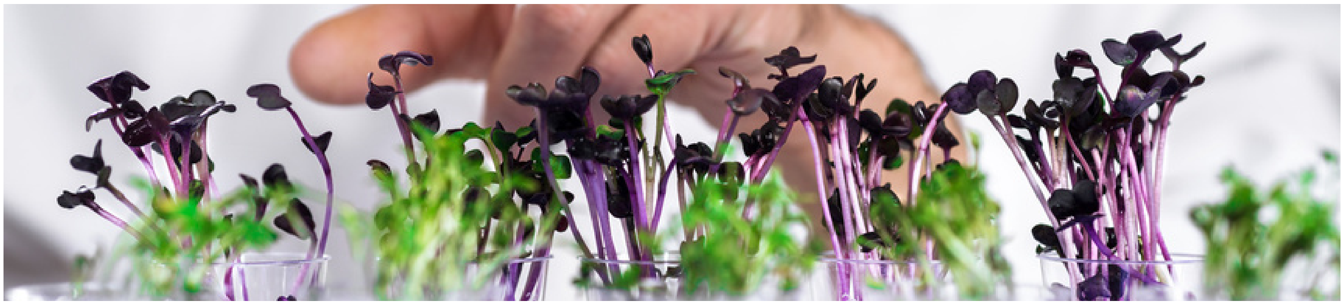

INVITED GUEST SPEAKERS

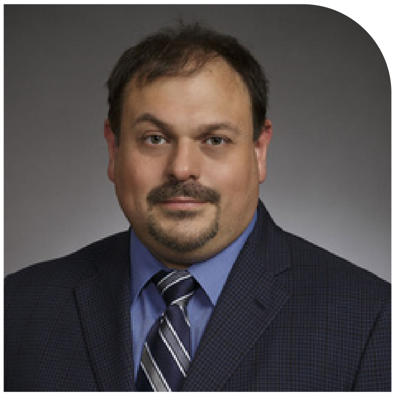

RONALD LORENZO, PHD

The Convergence of Social Movements: Social Justice, Open Science, and Community Science: the Challenges and Opportunities of Entering the Public Conversation

Ronald Lorenzo is an assistant professor of sociology at Prairie View A&M University, a public HBCU in Texas. He is an amateur molecular biologist through his participation in Eterna, an online research platform for RNA science and medicine based out of the Das Lab at Stanford University. Dr. Lorenzo is part of the Eterna development team. In addition to his sociological research in theory and culture, he has contributed as a community scientist to papers in RNA science. He is originally from San Jose, Costa Rica.

JOSH MORGAN, PSYD

Public Health Data for Whole Person Care

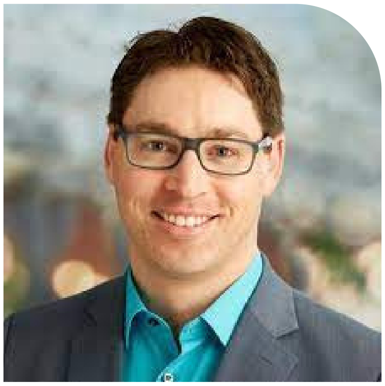

As SAS' National Director of Behavioral Health and Whole Person Care, Dr. Josh Morgan helps health and human services agencies use data and analytics to support a person-centered approach to improving health outcomes. A licensed psychologist, Dr. Morgan provides teletherapy through Marvin Behavioral Health, was previously San Bernardino County Department of Behavioral Health's Chief of Behavioral Health Informatics, is a member of the Board of Directors of Mental Health Services, a large non-profit community behavioral health provider in California, and is on the Advisory Board of the University of North Carolina's Center for Excellence in Community Mental Health. His clinical work includes adolescent self-injury, partial hospitalization, and intensive outpatient programs, psychiatric inpatient units and university counseling centers. Dr. Morgan earned his Bachelor of Arts in Religious Studies from the University of California, Berkeley, and a PsyD (Doctor of Psychology) in Clinical Psychology with an emphasis in Family Psychology from Azusa Pacific University, and is trained in Dialectical Behavior Therapy.

INVITED GUEST SPEAKERS

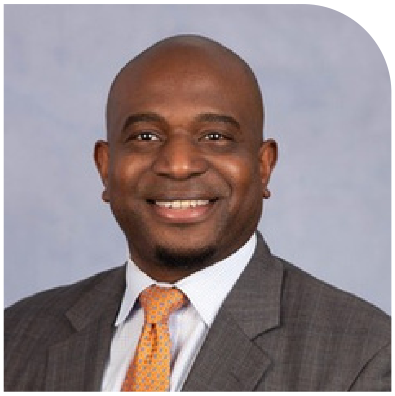

AZIZI SEIXAS, PHD

Technology and Innovation for Public Health

Dr. Azizi Seixas is a national and international thought leader in precision and personalized population health, digital health technology and innovation, and novel analytical tools like AI and machine learning to tackle our most vexing public health issues. He is currently Associate Professor of Behavioral Health and Psychology at the University of Miami Miller School of Medicine, Interim Chair of the Department of Informatics and Health Data Science, Director of The Media and Innovation Lab (The MIL), Associate Director of the Center for Translational Sleep and Circadian Sciences (TSCS) and leading the University of Miami's Digital Therapeutics vertical in the Institute of Data Science and Computing. He was selected as an Education Champion by Amazon Web Services for his innovative work using cloud computing technology and his advocacy for improving education and recognized by Cell Press, the world's most renowned scientific publishing house, as top 100 most inspiring Black scientists in America.

NANETTE STAR, MPH

Advancing Health and Human Services with GIS

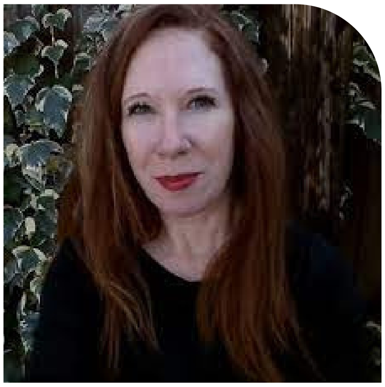

ESRI Health and Human Services Nanette Star is the Health & Human Services Industry Specialist at Esri. She is a strategic thinker and collaborator on all things data, health, and equity. She has over 15 years of experience in public and tribal health as an assistant director for a rural county public health department and a senior epidemiologist and project director with multiple tribal epidemiology centers. Nanette has also served as a public health policy coordinator for rural and tribal nations and has successfully evaluated and led policy, systems, and environmental initiatives with over 200 tribal nations and across five states. She holds a Master's degree in Public Health and Graduate Certificate in Epidemiology from George Mason University, as well as a BA in sociology and a minor in economics from California Humboldt State University. She is a certified yoga and meditation instructor and enjoys hiking and swimming with her golden doodles around northern California.

INVITED GUEST SPEAKERS

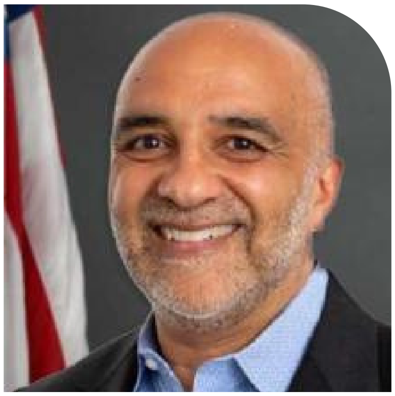

MICKY TRIPATHI, PHD, MPP

Updates from the National Coordinator for Health IT

Dr. Micky Tripathi, National Coordinator for Health Information Technology at the U.S. Department of Health and Human Services, will discuss enabling the 21st century digital healthcare system. Dr. Tripathi will discuss policies that will improve innovation in health care delivery, public health, and medical research including the Trusted Exchange Framework and Common Agreement (TEFCA), fulfilling a critical 21st Century Cures Act requirement and improving access to electronic health information. Plus, a look at what lies ahead for the Office of the National Coordinator for Health IT as it aligns federal health IT activities and works to fulfill a vision of better health enabled by data.

Need CE Credits?

Request Continuing Education Units for CPH, CAHIMS/CPHIMS, and/or CHES/MCHES by emailing **april@publichealthpodcasters.com.**

CAHIMS CPHIMS

INVITED GUEST SPEAKERS

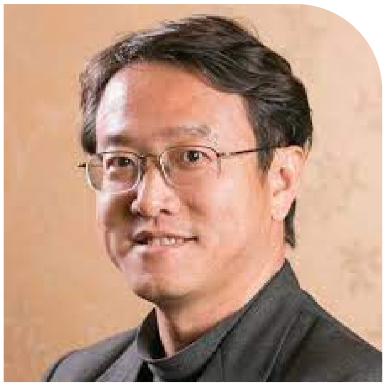

MING-HSIANG TSOU,
GIS and Health Workshop

Dr. Ming-Hsiang (Ming) Tsou is Professor of Geography, Director of the Center for Human Dynamics at the Mobile Age (HDMA), and the founding Program Director of Big Data Analytics Program (Master of Science) at San Diego State University. He received a Ph.D. (2001) from the University of Colorado at Boulder. His research interests are in human dynamics, social media, big data, public health, visualization, and Web GIS. Dr. Tsou has received over $4.39 million extramural funding, as PI or Co-PI, and $31 million funding as Co-Investigators, from NASA, NSF, NIH, USFS, and several state and local government agencies. He has published 106 refereed articles, three books, and one National Research Council Report (co-authored in 2007) since he started his academic career at SDSU in 2000. Dr. Tsou has collaborated with researchers at UCSD Moores Cancer Center, City of Hope, and Kaiser Permanente - Center for Health Research, to conduct GIS analysis for cancer research and to develop web- based GIS data visualization tools for studying health disparity of various cancers. He also collaborated with the County of San Diego Office of Emergency Services to develop an integrated wildfire evacuation decision support system by integrating social media, census survey, geographic information systems (GIS), real-time traffics, and remote sensing data. One of his recent research efforts focus on the social determinants of health in COVID-19 outbreaks and the mapping of vulnerable population and health disparity patterns in San Diego (https://hdma-sdsu.github.io/).

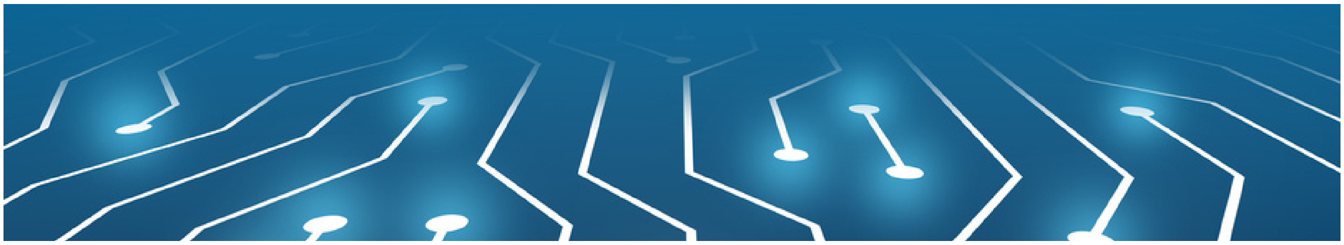

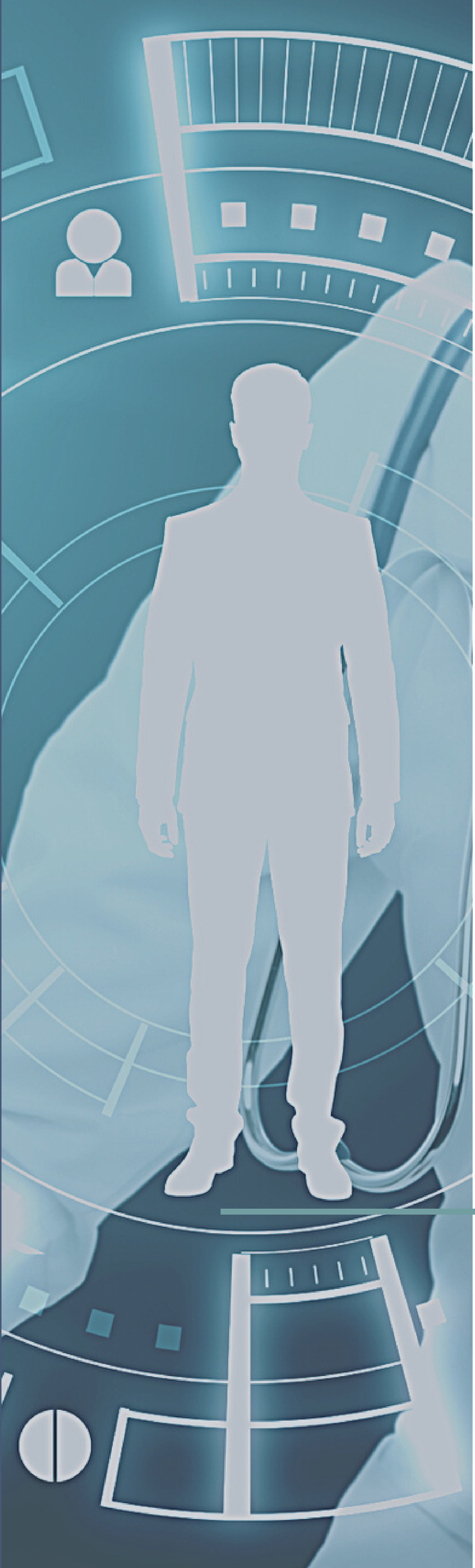

SPECIAL THANKS

STUDENT VOLUNTEERS

DIANA CARRILLO
MESA COLLEGE

JOSHUA CHOLA
UNIVERSITY OF LUSAKA

ALEMU DEBISO, PHD

HUAN-JU (COCO) SHIH
GEORGE MASON UNIVERSITY

VALERIA VALLEJO MONTOYA
UC SAN DIEGO

RAINACLARE SIBAL
UC BERKELEY

UNIVERSITY OF CALIFORNIA SAN DIEGO

INVITED GUEST SPEAKERS

PRESENTERS

ATTENDEES

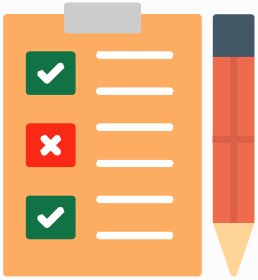

The PHIAT Conference welcomes your feedback at https://shorturl.at/giloR
